# Time depended Bcl-2 inhibition might be useful for a targeted drug therapy

**DOI:** 10.1186/s12935-015-0254-5

**Published:** 2015-11-02

**Authors:** Abdolhassan Talaiezadeh, Fateme jalali, Hamid Galehdari, Ali Khodadadi

**Affiliations:** Cancer, Environmental and Petroleum Pollutants Research Center, Ahvaz Jundishapur University of Medical Sciences, Ahvaz, Iran, Ahvaz, Islamic Republic of Iran; Department of Genetics, Shahid Chamran University of Ahvaz, Ahvaz, Islamic Republic of Iran; Department of Immunology, Ahvaz Jundishapur University of Medical School, Ahvaz, Islamic Republic of Iran

**Keywords:** Small interfering RNA, Apoptosis, Autophagy, Gene silencing, Nanoparticles

## Abstract

**Background:**

Over expression of Bcl-2 is frequently observed in several types of cancers and it is one of the prognostic markers in breast cancer. The importance of the Bcl-2 protein as ideal therapeutic target is the dual role of inhibiting apoptosis and autophagy-mediated cell death. Thus, the bcl-2 targeting may be a strategy of choice to improve treatment efficacy and overcome drug resistance to cancer chemotherapy. For this reason, we designed the siRNA mediated silencing of the Bcl-2 gene in the MCF-7 breast cancer cell line.

**Objectives:**

The purpose of this research was to investigate the effective Bcl-2 gene silencing by our homemade siRNA, more than previous study. Our data demonstrated that specific inhibition of the Bcl-2 by siRNA induces approximately more than 90 % gene silencing.

**Methods:**

MCF-7 Cell lines were treated by homemade Bcl-2siRNA for the first time and control siRNA that was transfected with nanoparticle. The cells harvested at 24, 48 and 72 h and transcription level of Bcl-2 was examined by Real Time -PCR analysis. The drug sensitivity was detected by using LDH assay test. Finally Anexin V-FITC test was performed for evaluation of apoptosis.

**Results:**

In the present study, results showed that targeting the specific sequence of the Bcl-2 by our homemade siRNA in the MCF7 cell line and its effect was more obvious in 24 h in contrast to 48 and 72 h.

**Conclusions:**

However, we showed here a time dependent blocking of the bcl-2 transcript that might lead to cell dead due autophagy, and not necessarily to apoptosis.

## Background

When cell is able to overcome a number of failsafe mechanisms, including apoptotic and autophagic cell death, and is also able to induce oncogene activation and tumor suppressor inactivation, it is said that the cell is cancerous [1]. Among women, breast cancer is the most frequently diagnosed cancer and the leading reason of cancer death, reported for 23 % of the total cancer cases and 14 % of the cancer deaths [2]. It is the most prevalent cancer both in the developed and developing countries.

Its development process involves decreasing expression of apoptosis gene and overexpression of anti-apoptosis and the genes involving in inhibition of autophagy such as BAD and Bcl-2, respectively [3–5].

Treatments include surgery, radiation therapy, chemotherapy [6]. While chemotherapy is an important therapy to breast cancer, the result of it in breast cancer is not ideal [3].

Overexpression of Bcl-2 is frequently observed in several types of cancer such as breast, lung, ovarian, melanoma cancers, and is often associated with unfavorable outcome [7–10]. So impairment of Bcl-2 gene expression is a hallmark of cancer and can result in resistance to chemotherapy [11, 12].

The important reason that creates Bcl-2 protein as an ideal therapeutic target is the dual role of it in inhibiting both apoptosis and autophagic-associated cell death [13, 14].

Apoptosis (self-killing) and autophagy (self-eating) are two self-destructive processes which have captured the attention of researchers over the last decades. While apoptosis includes the activation of catabolic enzymes leading to the destruction of cellular structures and organelles, autophagy is a slow, localized phenomenon which contains the sequestration of cytoplasmic constituents (including organelles and long-lived proteins) into double-membrane-bound vesicles or autophagosomes, which ultimately fuse with lysosomes for degradation [15, 16]. These pathways are two key signaling pathways employed by the cell in response to different inducers. The mechanisms of them are different, and involve basically diverse sets of regulatory and executioner molecules [17–19].

Bcl-2 suppress apoptosis by binding to Bax or Bak, and inhibit autophagy by binding to the protein Beclin 1, which is required for the initiation of autophagasome formation in autophagy [20–22]. Therefore, Bcl-2 not only functions as an anti-apoptotic protein, but also as an anti-autophagy protein via its inhibitory interaction with Beclin 1 [7].

So the crosstalk between apoptosis and autophagy is complex in nature, and sometimes inconsistent, but certainly acute to the overall fate of the cell [23].

It is proposed that functional blockage of the anti-apoptotic Bcl-2 gene could possibly change the balance of the apoptotic and autophagy machinery in tumor cells and sensitizes them to chemo and radiotherapy. Thus, targeting of Bcl-2 may be a strategy of choice to improve treatment efficacy and overcome drug resistance to cancer chemotherapy. Small interfering RNA (siRNA) is also a powerful tool to validate the targets of therapeutic drugs. RNAi is being explored as a powerful tool to inhibit the expression of genes involved in oncogenes and genes that are involved in angiogenesis, metastasis, survival, anti-apoptosis and resistance to chemotherapy [24, 25]. And it has shown great promise for many diseases such as cancer.

The silencing effect of siRNAs isn’t long-lived, since the siRNAs ultimately decay within the cell [25], and this can be an advantage because this method is used to increase the efficiency of chemotherapy which is done in a short period of time.

Synthetic siRNAs strongly inhibit expression of the target gene in mammalian cells when they are transfected into the cells by cationic liposomes [26].

## Objectives

Resistance to chemotherapeutic agents is a major factor confounding cancer treatment. siRNAs have been used to decrease the drug resistance of cells in vitro by inhibiting the expression of Bcl-2. The degree of siRNA-mediated gene silencing was consistent with other reports where antisense reagents of several types were used to target Bcl-2 [27–30].

The purpose of the study was to investigate the possibility of Bcl-2 gene silencing by our homemade siRNA.

## Methods

### Cell calture

As Bcl-2 is an effective target for gene silencing in MCF-7 breast cancer cells, this cell line was obtained from Pasteur Institute of Iran. And then was cultured in RPMI-1640 medium containing 10 % fetal bovine serum (FBS) and 1 % penicillin–streptomycin under standard culture conditions (37 °C, 95 % humidified air and 5 % CO2).

### Transfection of siRNA

First, in six-well tissue culture plates containing 2 mL antibiotic-free normal growth medium supplemented with FBS, 2 × 10^5^ cells per well were seeded. The cells were then Incubated at 37 °C in a CO_2_ Incubator until they were 60–80 % confluent (This takes 18–24 h). Then the siRNA duplex solution was then added directly to the dilute Transfection Reagent using a pipette and mixed gently by pipetting the solution up and down. The mixture were incubated at room temperature for 15–45 min, and washed with 2 mL of siRNA transfection medium.

For each transfection, 0.8 mL of siRNA transfection medium was added to each tube containing the siRNA transfection reagent mixture, and was mixed gently and covertly the mixture on to the washed cells. The cells were then incubated in a CO_2_ incubator at 37 °C for 5–7 h.

**Fig. 1:**
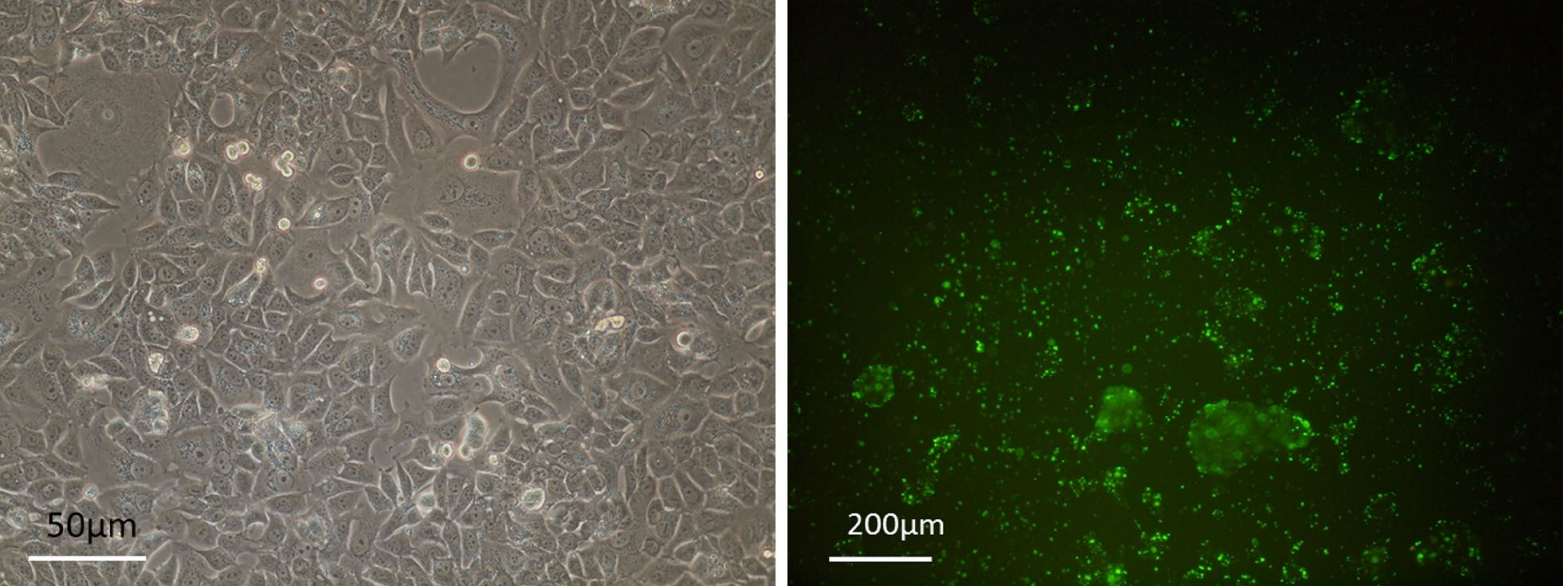
Experiment after a 6-h of treatment with the control siRNA. Uptake of FITC-labelled siRNAs of Bcl-2 in MCF-7 cells, verified by fluorescence microscope

**Fig. 2:**
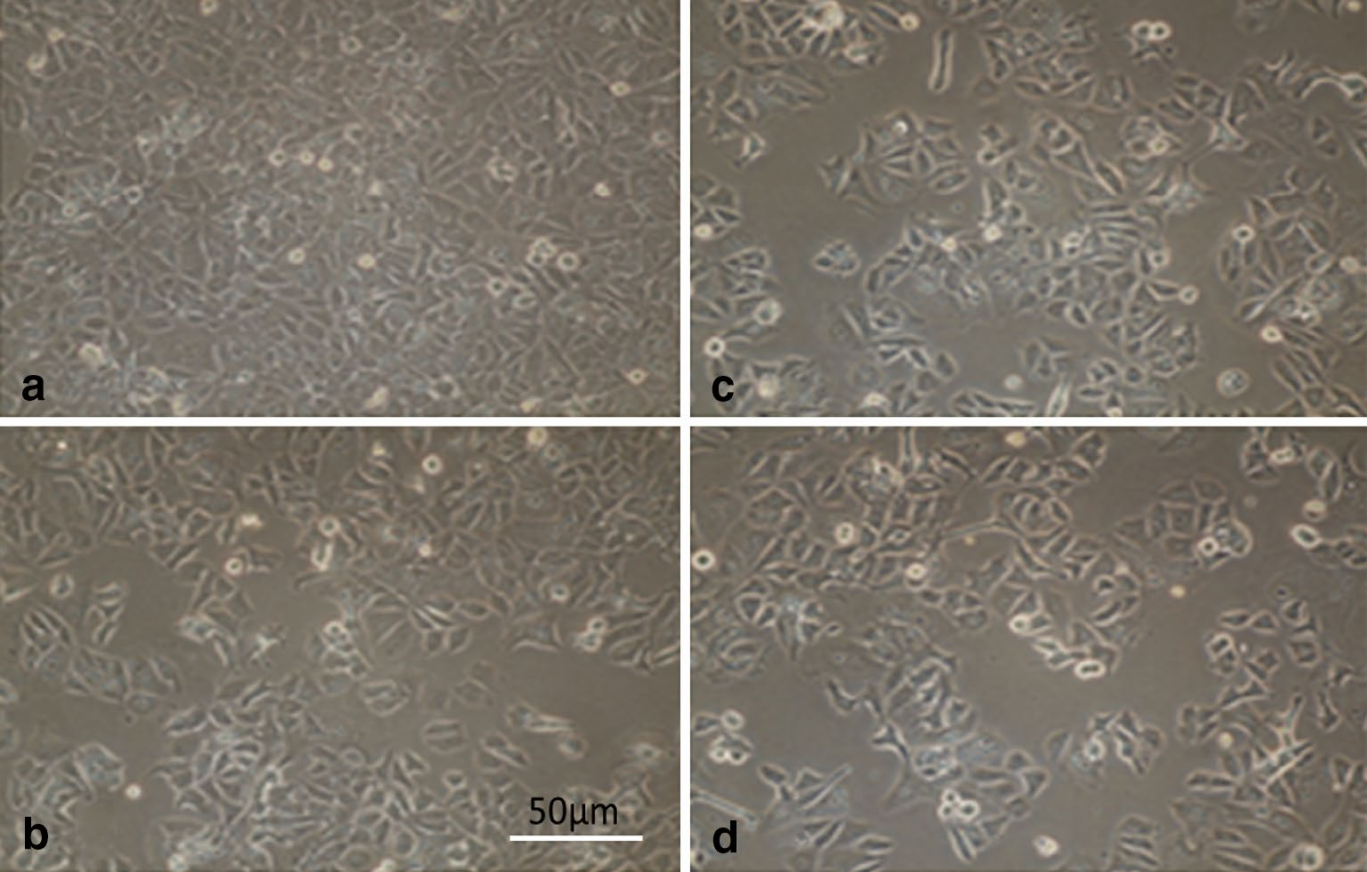
**a** Normal cell line. **b** 24 h after transfection. **c** 48 h after transfection. **d** 72 h after transfection. Probably unknown agent has affected cell line viability in 48 h after transfection

The siRNA sequence which was supposed to targete bcl-2 was designed by Genescript, and is shown below: Bcl2sense:5′-UCAGUAGGUGUCCCGCUACTT-3′ bcl-2 antisense: 5′-TTAGUCAUCCAC AGGGCGAUG-3′. To determine that our homemade siRNA is the effector molecules of the RNAi pathway, we used control (no targeting) siRNA duplexes which have four or more mismatches with all known human mRNAs, and have no homology with any known gene (Fig. 1). The cells were transfected with siRNA according to the manufacturer’s instructions. On the day of transfection, the medium was replaced by 2 mL of fresh medium and 1 μg of siRNA, and then was mixed with the transfection reagent. All experiments were performed at 24, 48 and 72 h post transfection. Finally cells were harvested for analysis 72 h after transfection. For enhancing the transfection efficiency, the cells were transfected with the non-silencing FITC-labeled siRNA for 4 h, washed with phosphate-buffered saline twice, and counted in a fluorescent microscope (Figs. 1 and 2). After treatments, the cells were harvested at different time including 24, 48 and 72 h for real-time analysis and for evaluation of apoptosis and autophagy state as well.

### Total RNA extraction and cDNA synthesis

Total cellular RNA was isolated using the RNeasy Mini Kit (Qiagen, Valencia, CA). According to manufacturer’s instructions and then was quantified by nanodrop.

cDNA synthesis was performed using BIONER cDNA Synthesis Kit. And then cDNA was verified by PCR using gene specific primers for GAPDH as an internal control.

### Real-timePCR

The sequences of Real-Time reverse transcription-polymerase chain reaction amplification of Bcl-2, GAPDH cDNA, and Bcl-2 primers were (5′-GGAGAGTGCTGAAGATTGATG-3′), (5′-AGTCTACTTCCTCTGTGATGTTG-3′), for forward and reverse respectively.

GAPDH primers were (5´-AGGGCTGCTTTTAACTCTGGT-3´), (5´-CCCCACTTGATTTTGGAGGGA -3′) for forward and reverse respectively.

### Analysis of apoptosis

To provide a comparative assay of apoptosis by Annexin V labeling, tumor cells (1 × 10^6^) were treated with Bcl-2 siRNA for 24–72 h and then were harvested and washed with PBS. Cells were resuspended in binding buffer and stained with Annexin V and propidium iodide (PI) according to manufacturer’s protocol (Abcam Kit), control and treated cells were detected and quantified by FACS analysis.

### Lactate dehydrogenase activity-based cytotoxicity Assays

Non-radioactive colorimetric assay is suitable for high-throughput quantification of cell death and cell lysis, based on measurement of lactate dehydrogenase (LDH) activity released from the cytosol of damaged cells. To do this, we let the cells proliferate, and at the end of experiment, the cells were lysed by adding the lysis reagent supplied with the kit. Total released LDH was measured with the reaction mix from the kit, according to the manufacturer’s protocol (Cytotoxicity Detection Kit PLUS (LDH) Roch).

### Statistical analysis

Statistical analysis was carried out by one-way ANOVA.In all other cases, the results from at least three individual experiments (*n* ≥ 3) were averaged to determine mean and statistically significant difference.

## Results

In combination with standard chemotherapy, treatment with siRNA can also decrease the chemo resistance of specific cancers, indicating the potential of siRNA therapy for treating many malignant diseases [25]. Two strategies must be carefully considered to address safety concerns and to guarantee effective, successful treatment of human diseases are design and delivery for RNAi effector molecule [31]. So screening candidate siRNA for homology with available sequence databases can, in principle, predict and avoid several off-target effects we had predicted that our desined siRNA has more than 80 % efficiency.

Delivery is probably the only biggest obstacle to the development of RNAi-based therapeutic agents. Liposomes are commonly used carriers, delivering the siRNA with better transfection efficiency and protecting it from degradation [25] Yano et al. have used such a liposome to show that anti-bcl-2 siRNA complexed with cationic lipid liposome had a strong antitumor activity when executed intravenously in the mouse model of liver metastasis [32]. So in our study the cell line was transfected using siRNA Transfection Reagent (Santa Cruz Biotechnology, Inc), that is a cationic lipid which its components are the size of Nano particle that it cause more transfection efficiency.

Control siRNA (FluoresceinConjugate)-A is recommended for measuring transfection efficiency of cationic lipid based transfection reagents in cells, in order to determine the most suitable transfection reagent to utilize for RNAi studies.

### Down regulation of Bcl-2 mRNA expression in MCF-7 breast cancer cells

Specific down regulation of Bcl-2 by antisense oligos or siRNA sensitizes cancer cells to chemotherapy or radiation therapy. Hence to inhibit the expression of Bcl-2, we applied siRNA to target directly the Bcl-2 transcript. We studied time-dependent down regulation of Bcl-2 and observed that Bcl-2 siRNA-induced down regulation of Bcl-2 mRNA, was started at 24 h after treatment but an unknown cause led to increase in gene expression of Bcl-2 in 48 h after transfection. Interestingly, most of the gene silencing of Bcl-2 was observed in 24 h after transfection of siRNA, and it is unlike the previous studies.

Down regulation of Bcl-2 mRNA level was observed after 48 h. Interestingly, after 72 h Bcl-2 gene expression level came back to normal level. Nevertheless, down regulation of Bcl-2 transcription level in 24 h was more than other periods.

### LDH assay test showed no significant sign of cell death

LDH test indicated higher apoptosis in cell treated with siRNA in different times in contrast to controls but the results were not approved by statistical analysis (no data shown).

### Bcl-2 siRNA decrease apoptosis cell death in MCF-7 cell line

We next evaluated programmed cell death mechanisms, including apoptosis. One of the earliest changes during apoptosis is translocation of the membrane phospholipid phosphatidyl serine from the inner leaflet of the membrane to the outer one. Therefore, phosphatidyl serine expression at the external membrane was investigated by the Annexin V assay [33, 34].

No increase in apoptosis was found in MCF-7 cell line treated with Bcl-2 siRNA evaluating by Annexin staining at 72 h. A time-dependent decrease in apoptosis of MCF-7 cell line was associated with down regulation of Bcl-2 mRNA. These findings suggest that apoptosis inhibition by down regulation of Bcl-2 might not play an important role in cell death, and that cell death was probably due autophagy.

## Discussion

We aimed here to appropriate blocking of the bcl-2 function as a potent apoptosis trigger by siRNA silencing. On transcription level we observed a time depended and significant decreasing of the bcl-2 mRNA. But this event was restricted for 24 h. However, in this sight of view, it could be the efficient time for appropriate drug therapy against chemotherapy resistant tumor cells. The other hallmark of the present study was the improvement of the idea that the bcl-2 blocking would not necessarily lead to apoptosis, but to autophagy, as will be discussed.

**Fig. 3:**
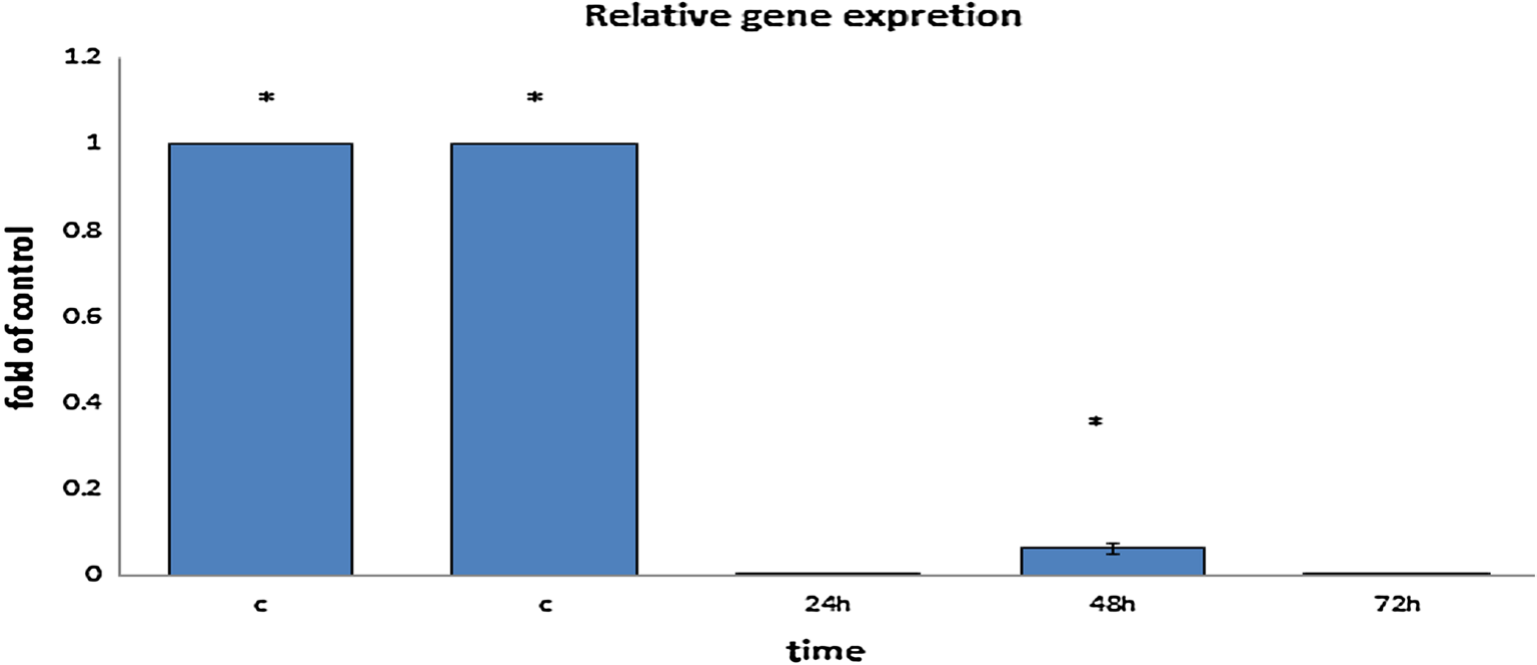
Down regulation of Bcl-2 mRNA after 24, 48 and 72 h of treatment with Bcl-2 siRNA gene silencing

The first silencing of the bcl-2 gene by RNAi in MCF-7 cells was reported by RT Lima et al. in 2004. They showed that down regulation of bcl-2 leads to increase of spontaneous apoptosis but at the same time they used new designed siRNA targeting gene expression of the xIAP inhibiting caspases inhibitors downstream of Bcl-2 [28]. In 2005, Buchholz TA found that down regulation of the Bcl-2 in the MCF-7 cells by siRNA induces cell death 50 % above control. However, apoptosis has been counted for just 11 % of cell death. They further suggested autophagy as possible alternative cell death. In 2008 Akar u and et al. provided the first evidence that targeted silencing of Bcl-2 by siRNA induces autophagic cell death in breast cancer cells with overexpression of Bcl-2. Their findings support the idea that Bcl-2 plays a crucial role in inhibition of breast cancer cells from autophagic cell death [35]. Our results indicated that the homemade siRNA induces effective Bcl-2 gene silencing in MCF7 cell after 24 h treatment (Fig. 3). But after 48 h of treatment, expression reduction ceases and rises in contrast to 24 h and after 72 h the expression level came back to normal range. This finding is in relation to the previously reported results by Akar U et al. using Real-time PCR and indicates that our homemade siRNA can successfully silence the target gene. Further investigation of the Bcl-2 expression by Real-time PCR or AnnexinV in the present report yielded similar results and approximately confirmed each other.

Bcl-2 protein interacts with Beclin 1 protein which is an autophagy factor, and Bcl-2 reduction causes Beclin 1 to release, which triggers autophagy. Apoptosis and autophagy are two separated mechanism. It has been reported that autophagy blocks apoptosis in some cases. To interpret our results, reduction in apoptosis after 24 h of treatment with siRNA is due to autophagy activation, and sudden reduction of apoptosis after 48 h in contrast to 24 and 72 h is probably because of anti-apoptosis characteristics of Bcl-2 gene, which results in apoptosis reduction.

**Fig. 4:**
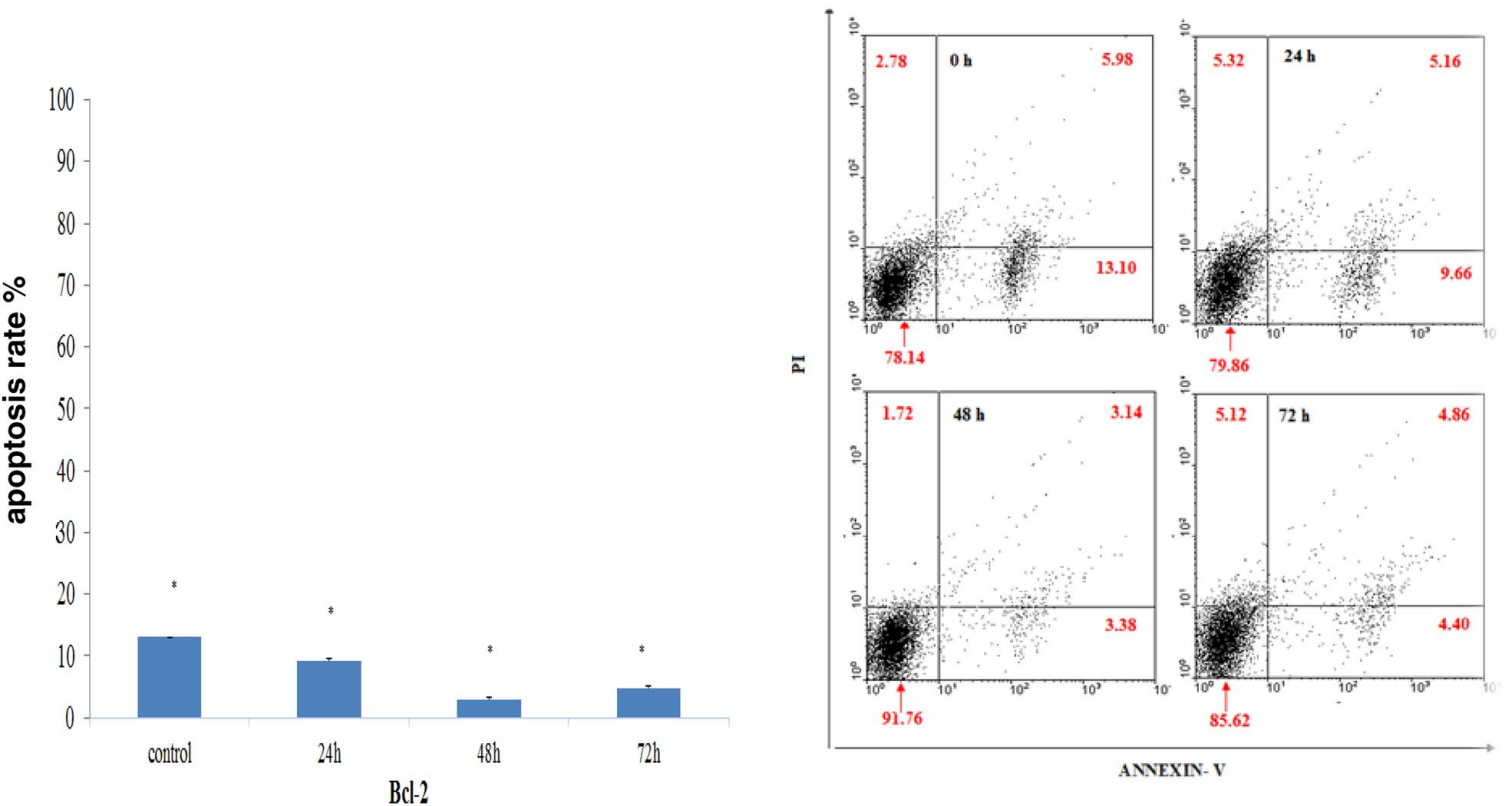
Treatment with Bcl-2 siRNA does not induce apoptosis in MCF-7 breast cancer cells. MCF-7 cells were stained with Annexin V after the treatments at 72 h, and positive cells were quantified by FACS analysis as described in “Methods”

Based on the fact that the role of autophagy in cell survival and cell death is debatable, and it is double-edged sword, If we assume autophagy occurence by other incidences, Bcl-2 silencing probably activates cell survival mechanism of autophagy that acts as an adaptive response to provide nutrients and energy by breaking down cellular building blocks such as mitochondria and golgi statue, an active bax is localized at the trans-golgi network and is one of the main membranes of apoptosis, So it may lead to loss of the internal pathway of apoptosis. It is probably one of the reasons for decrease in apoptosis in AnexinV test results (Fig. 4).

We tried to illustrate conflicting results that observed at 48 h after transfection, but as many intracellular molecular mechanisms are still unknown furthermore, to clarify the relationship between different types of cell death, more study is needed, and must provide additional tools for analysis these processes.

Also an unfortunate irony of the revolution in understanding cancer as a genetic disease is that a single accepted treatment directly targets the products of the genes that are most frequently activated in human cancers without interfering with other pathways in human cancers [36]. Although quite a lot of studies have been done on Bcl-2, there are numerous unclarified interactions partners which regulate its activities and connect it to a wide variety of cellular pathways.

Although our study confirmed the previous studies that Bcl-2 gene silencing doesn’t induce apoptosis [35, 36], it remains an ongoing mystery how the cells ‘decide’ to respond to siRNA by preferentially undergoing autophagy, and not apoptosis.

In conclusion, our results indicated the possibility of complete down regulation of the Bcl-2 mRNA level by novel designed Bcl-2 in the MCF-7 human breast cancer cells. But, the question remains whether the cell dead is due apoptosis or autophagy or even a combination of both pathways. However, studies at the molecular level on the interplay between autophagy and apoptosis are necessary. To explore the sensitization effects of gene silencing, Bcl-2 siRNA should also be combined with chemotherapeutic agent.
